# Species composition and richness of aphid parasitoid wasps in cotton fields in northern China

**DOI:** 10.1038/s41598-017-10345-7

**Published:** 2017-08-29

**Authors:** Fan Yang, Yue-Kun Wu, Lei Xu, Qian Wang, Zhi-Wen Yao, Vladimir Žikić, Željko Tomanović, Mar Ferrer-Suay, Jesús Selfa, Juli Pujade-Villar, Yan-Hui Lu, Yu-Yuan Guo

**Affiliations:** 1 State Key Laboratory for Biology of Plant Diseases and Insect Pests, Institute of Plant Protection, Chinese Academy of Agricultural Sciences, Beijing, 100193 China; 2 Faculty of Sciences and Mathematics, Department of Biology and Ecology, University of Niš, Višegradska 33, 18000 Niš, Serbia; 3 University of Belgrade, Faculty of Biology, Institute of Zoology, Department of Invertebrate Zoology and Entomology, Belgrade, 11000 Serbia; 4 Universitat de València, Facultat de Ciències Biològiques, Departament de Zoologia, València, 46100 Spain; 5 Universitat de Barcelona, Facultat de Biologia, Departament de Biologia Animal, Avda. Diagonal 645, 08028 Barcelona, Spain

## Abstract

The cotton aphid, *Aphis gossypii* (Hemiptera: Aphididae), is a serious pest of cotton across the globe, particularly in the cotton agroecosystems of northern China. Parasitic wasps are deemed to be important natural enemies of *A*. *gossypii*, but limited information exists about their species composition, richness and seasonal dynamics in northern China. In this study, we combine sampling over a broad geographical area with intensive field trials over the course of three cropping seasons to describe parasitoid-hyperparasitoid communities in cotton crops. We delineate a speciose complex of primary parasitoids and hyperparasitoids associated with *A*. *gossypii*. Over 90% of the primary parasitoids were *Binodoxys communis*. *Syrphophagus* sp. and *Pachyneuron aphidis* made up most of the hyperparasitoids. Parasitism rates changed in a similar way following the fluctuation of the aphid population. Early in the growing period, there were more hyperparasitoids, while later, the primary parasitoids provided control of *A*. *gossypii*. The first systematic report of this cotton aphid parasitoid complex and their population dynamics in association with their hosts presented a comprehensive assessment of cotton parasitoid species and provided important information for the establishment and promotion of their biological control of cotton aphids.

## Introduction

Aphids cause economic losses in many crops worldwide. In many cropping systems, parasitoids play a central role in the control of aphid pests. Diverse parasitoid communities, composed of both primary and secondary parasitoids, establish intricate trophic relationships with their aphid hosts and associated host plants. For example, 13 species of Aphidiinae (Hymenoptera: Braconidae) parasitoids from seven genera are associated with aphids on pome and stone fruit trees, establishing as many as 69 different tritrophic associations^1^. On alfalfa crops in Spain, up to 13 species of aphidiine wasps and four species of aphelinids have been found as well as eight hyperparasitoids^2^. Lastly, cereal aphids are subject to parasitism rates of 30–80% on Danish and New Zealand winter wheat, mostly from *Aphidius ervi* Haliday and *Aphidius rhopalosiphi* De Stefani-Perez^3^.

Hyperparasitoids that oviposit eggs directly into the eggs or larvae of primary parasitoids inside a premummified aphid are called true hyperparasitoids, such as *Alloxysta* (Cynipidae)^4^, while species of *Asaphes* and *Pachyneuron* (Pteromalidae) and *Dendrocerus* (Megaspilidae), which feed externally on the primary or secondary larval parasitoids inside the mummies, are mummy parasitoids^5^. Hyperparasitoids may disrupt primary parasitoids’ limited biological control of aphid populations^6^. The percentage of hyperparasitism of *Lysiphlebus hirticornis* Mackauer in colonies of *Metopeurum fuscoviride* (Stroyan) aphids can reach over 50% and causes significant mortality among primary parasitoids^7^. However, successful biological control has been reported in the presence of significant hyperparasitism^8–10^. The overall impact on aphid and parasitoid populations might be explained by low average fecundity^7^. Overall, the function and influence of hyperparasitoids, the top consumers in this system, on aphids and parasitoids is complicated.

The cotton aphid, *Aphis gossypii* Glover, is a major pest of cotton worldwide^11^. In northern China, this pest primarily affects cotton crops in the early growth stages^12–14^. Its climatic adaptability, reproductive ability, and capacity to rapidly attain high field populations contribute significantly to its pest status. Although *A*. *gossypii* directly impacts cotton yields through sap feeding and honeydew secretion, it also acts as an efficient vector of multiple plant viruses, such as cucumber mosaic virus (CMV) and others^15, 16^. Although parasitoids are important natural enemies of *A*. *gossypii*, parasitism rates tend to remain below 30%^17–21^. The dynamics of parasitoid wasp populations were only presented with the abundance of aphid mummies^22, 23^, mummification rates^20^ or Aphididae and hyperparasitoid proportions^18^. Cotton aphid parasitoids mainly belong to Aphidiinae (Braconidae) and Aphelinidae, with the former subfamily including *Aphidius*, *Binodoxys*, *Lipolexis*, *Lysiphlebia*, *Lysiphlebus* and *Trioxys* species. Among these, *Lysiphlebia japonica* (Ashmead) has received most of the scientific attention^18, 21, 24–29^. Aphelinidae are represented by *Aphelinus asychis* Walker^30^ and *A*. *basilicus* Fatima & Hayat^31^. In addition to the above groups of primary parasitoids, hyperparasitoids can play a prominent role in shaping aphid-parasitoid trophic interactions in cotton agroecosystems. However, little or no attention has been paid to hyperparasitoids in cotton crops around the world or within China. In eastern China, a total of four hyperparasitoid species have been reported in cotton fields, including *Lygocerus koebelei* Ashmead (Hym., Ceraphronidae) and *Syrphophagus* sp. (Hym., Encyrtidae)^18^. However, up to now, no comprehensive study has been conducted regarding the primary and secondary parasitoid species associated with *A*. *gossypii* in northern China.

In this study, we describe the parasitoid communities associated with *A*. *gossypii* in northern China. More specifically, we record 1) the species diversity and relative abundance of both primary and secondary parasitoids of *A*. *gossypii*, and 2) the seasonal aphid × parasitoid dynamics. Our work helps determine the importance of parasitoid-mediated biological control of *A*. *gossypii* in northern China and lays the necessary groundwork for the subsequent development of biological control tactics for conservation.

## Results

### Study #1: Species composition

In 2014, a total of 1,448 parasitoid specimens were collected, which included two primary parasitoid species (72.2%), *Binodoxys communis* (Gahan) and *Aphidius gifuensis* (Ashmead), and ten different hyperparasitoid species (27.8%), *Alloxysta brevis* (Thomson), *A*. *pusilla* (Kieffer), *Asaphes suspensus* (Nees), *Dendrocerus laticeps* (Hedicke), *Pachyneuron aphidis* (Bouché), *Phaenoglyphis villosa* (Hartig), *Syrphophagus aphidivorus* (Mayr), *S*. *eliavae* Japoshvili, *S*. *taeniatus* (Förster) and *Syrphophagus* sp. In 2015, a total of 2,901 parasitoid specimens were collected, including the same ten hyperparasitoid species (62.7%) as 2014, *B*. *communis* and a different primary parasitoid species, *Aphelinus albipodus* Hayat and Fatima (37.3%).

**Figure 1:**
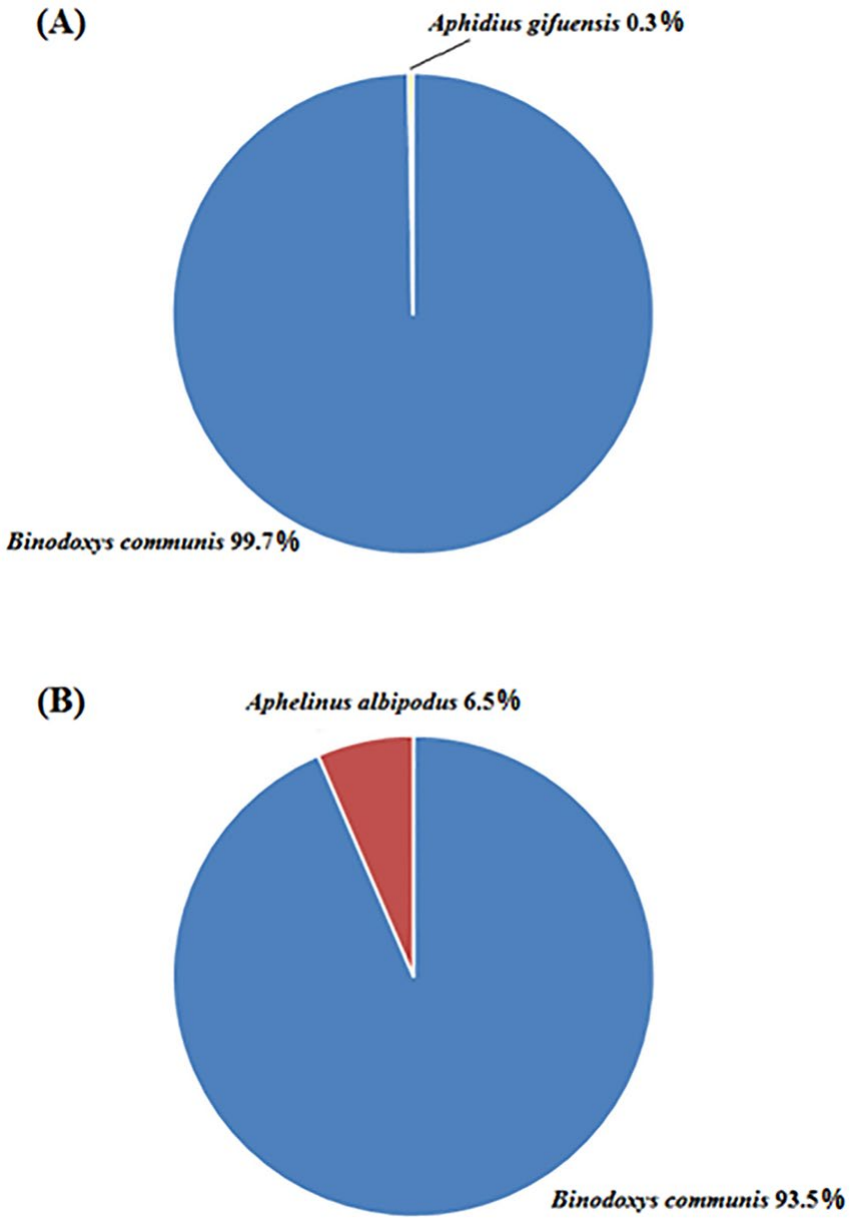
Composition of the primary parasitoids associated with the cotton aphid, *Aphis gossypii*, in cotton fields in northern China in 2014 and 2015. (**A**) Composition in 23 cotton fields in 2014 and (**B**) composition in 16 cotton fields in 2015.

**Figure 2:**
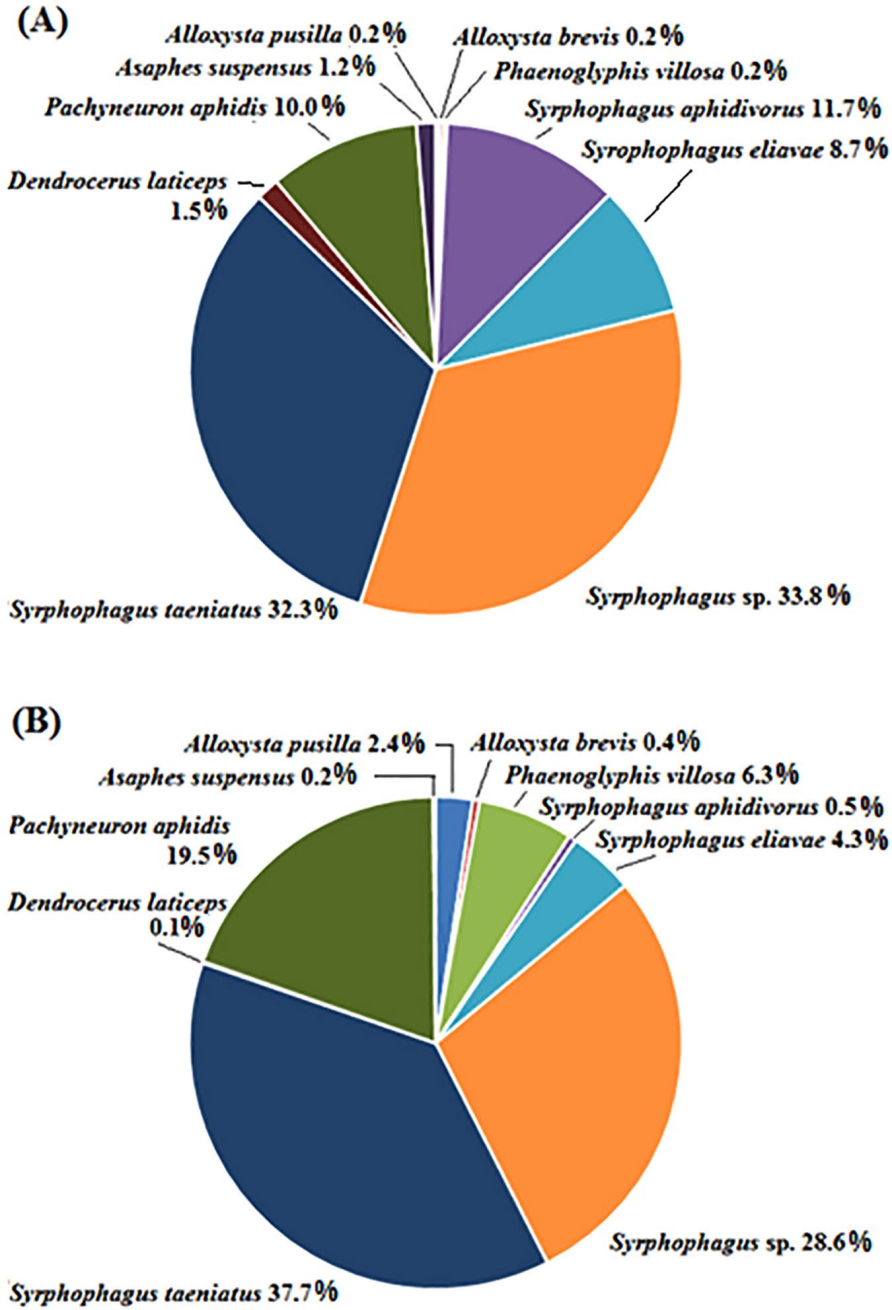
Composition of the hyperparasitoids of the cotton aphid, *Aphis gossypii*, in cotton fields in northern China in 2014 and 2015. (**A**) Composition in 23 cotton fields in 2014 and (**B**) composition in 16 cotton fields in 2015.

Of the primary parasitoids sampled in 2014 and 2015, 99.7 and 93.5%, respectively, were *B*. *communis*. In 2015, *A*. *albipodus* made up 6.5% (n = 40) of the primary parasitoids. For the hyperparasitoids, the same species were found in similar relative proportions with little differences across years, like *A*. *brevis* (0.2% for 2014, 0.4% for 2015), *Syrphophagus* sp. (33.8% for 2014, 28.6% for 2015), *S*. *taeniatus* (32.3% for 2014, 37.7% for 2015) *etc*. In 2014, *Syrphophagus* sp. was the most abundant hyperparasitoid, at 33.8%, followed by *S*. *taeniatus* (32.3%), *S*. *aphidivorus* (11.7%), and *P*. *aphidis* (10.0%). The remaining species collectively accounted for < 10% of all hyperparasitoids collected. In 2015, the most abundant hyperparasitoids were *S*. *taeniatus* (37.7%), *Syrphophagus* sp. (28.6%) and *P*. *aphidis* (19.5%) (Fig. 1, 2). Photographs of the main parasitoids and their key morphological features can be found in the Supplemental information (S1).

### Study #2: Aphid-parasitoid seasonal dynamics

**Figure 3:**
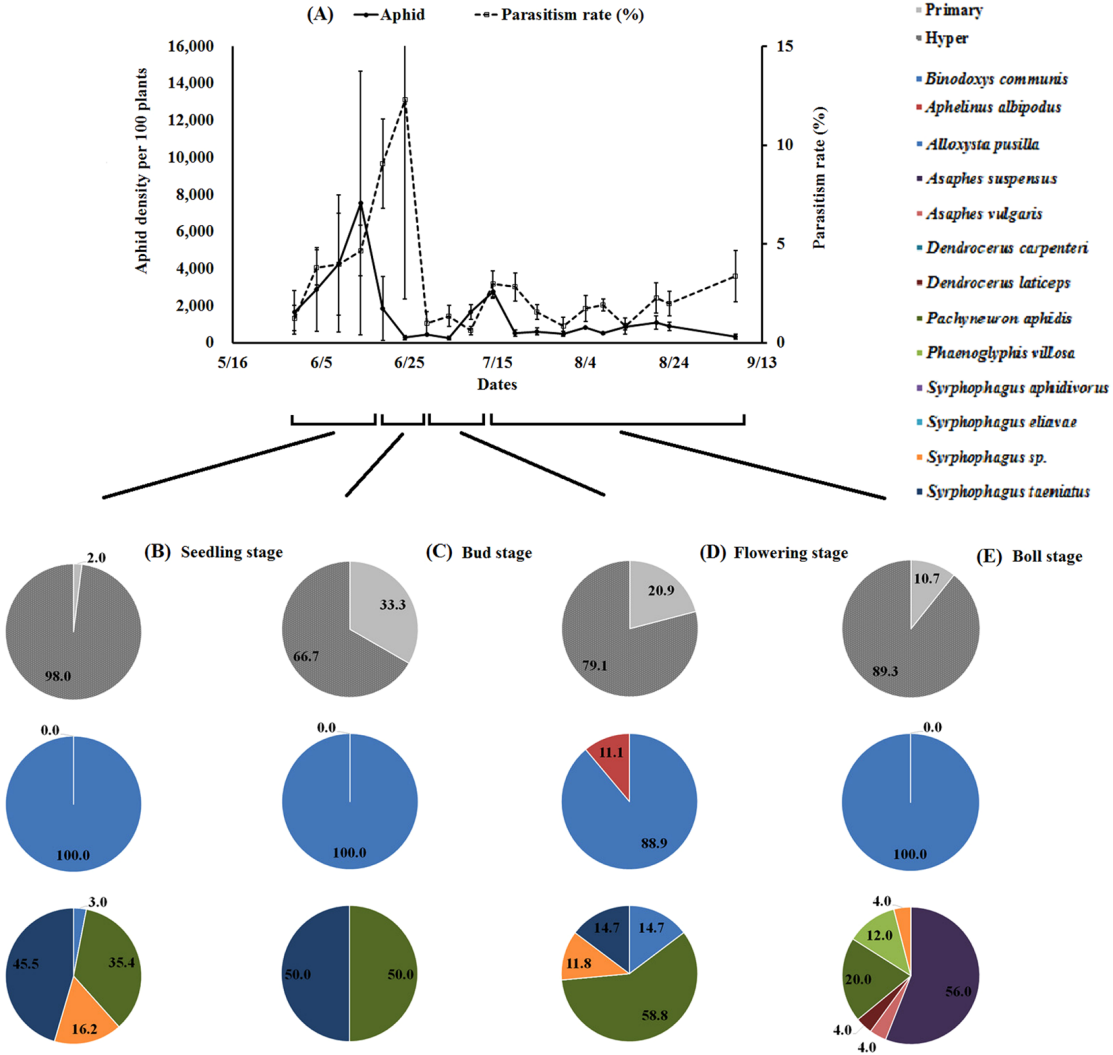
Cotton aphid, *Aphis gossypii*, density and parasitism dynamics (**A**), the proportion of cotton aphid primary parasitoid and hyperparasitoid diversity and composition at the seedling stage (5/30-6/19) (**B**) and at the bud stage (6/20-6/29) (**C**), the flowering stage (6/30-7/14) (**D**) and the boll stage (7/15-8/23) (**E**) in 2015 in cotton fields in Langfang, Hebei, China.

**Figure 4:**
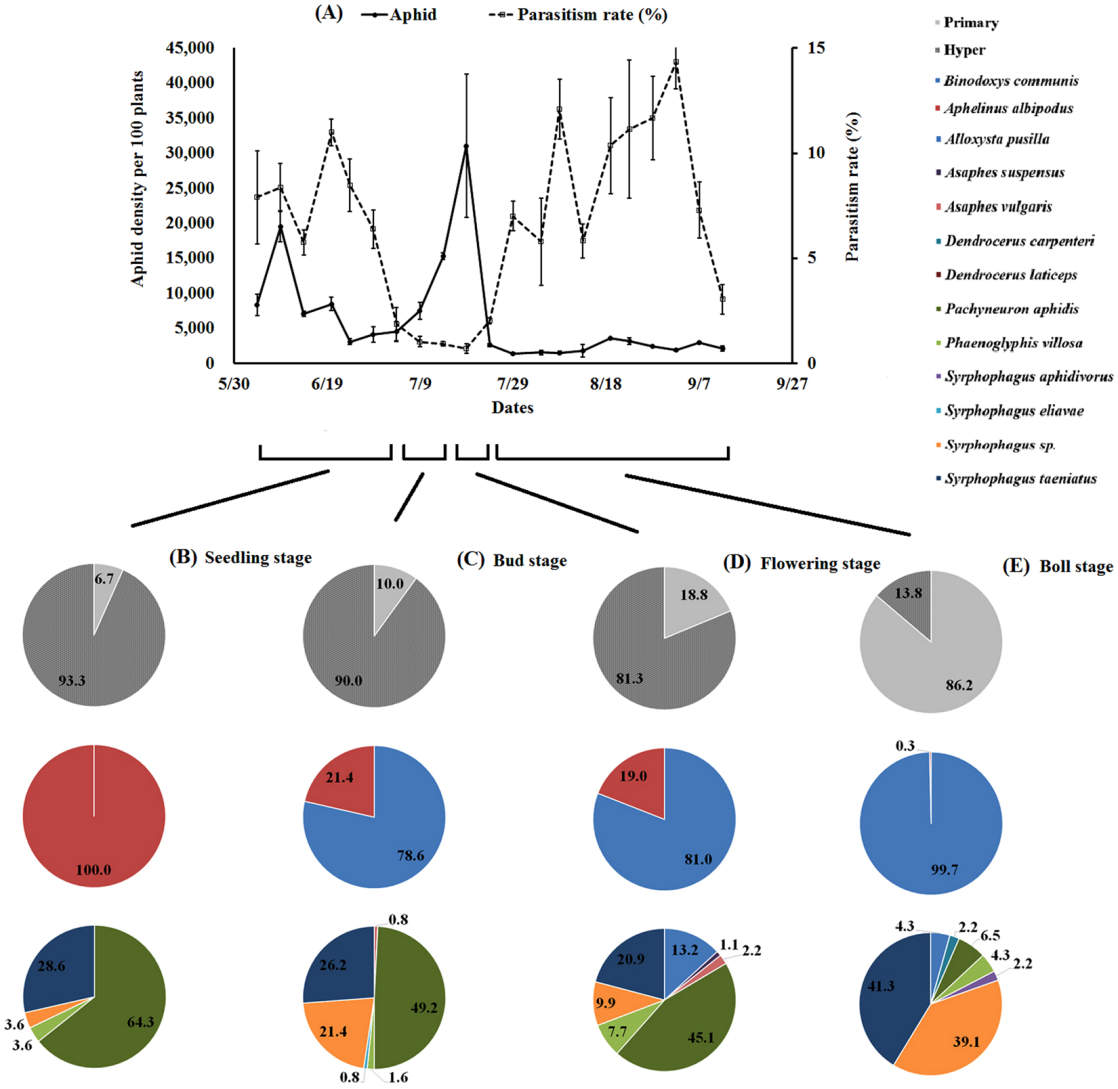
Cotton aphid, *Aphis gossypii*, density and parasitism dynamics (**A**), the proportion of cotton aphid primary parasitoid and hyperparasitoid diversity and composition at the seedling stage (6/4-7/4) (**B**) and at the bud stage (7/5-7/14) (**C**), the flowering stage (7/15-7/24) (**D**) and the boll stage (7/29-9/12) (**E**) in 2016 in cotton fields in Langfang, Hebei, China.

In 2015, *A*. *gossypii* populations increased from late May and reached an initial peak on June 14^th^, with 7,553 aphids per 100 plants, and a second peak on July 14^th^ (Fig. 3). In 2016, the *A*. *gossypii* populations reached an initial peak on June 9^th^ and then a maximum on July 19^th^, with a density of 31,029 individuals per 100 plants. On July 24^th^, the density suddenly dropped to 2,615 and then underwent only small fluctuations thereafter (Fig. 4).

Parasitoid populations lagged behind the *A*. *gossypii* build-up patterns. In 2015, parasitism rate peaked at 12.3% on June 24^th^ (Fig. 3). In 2016, peak parasitism levels were recorded on June 20^th^, August 8^th^ and September 2^nd^, with parasitism rates above 10% from August 19^th^ to September 2^nd^ (Fig. 4).

In Langfang, *B*. *communis* and *A*. *albipodus* were the two dominant primary parasitoids in both 2015 and 2016. Eight hyperparasitoid species were found in 2015 and ten in 2016. Compared to study #1, one additional hyperparasitoid species was found, i.e., *Dendrocerus carpenteri* (Curtis).

For the percentages of primary parasitoids and hyperparasitoids, there were significant differences throughout the whole growing period in both years (2015: χ^2^ = 16.49, df = 3, *P* = 0.0009; 2016: χ^2^ = 330.35, df = 3, *P* < 0.0001). In 2015, the hyperparasitoids dominated the parasitoid complex, with the highest percentage of 98.0% at the seedling stage and the lowest percentage of 66.7% at the bud stage, while the percentage of hyperparasitoids gradually decreased from 93.3% to 13.8% in 2016. *A*. *albipodus* only appeared at the flowering stage, with a percentage of 11.1%, and no significance difference was found throughout the growing period in 2015 (χ^2^ = 0.71, df = 3, *P* = 0.8698). However, the percentages of the two primary species were significantly different in 2016 (χ^2^ = 103.97, df = 3, *P* < 0.0001), and *B*. *communis* increased from 0.0% at the seedling stage to 99.7% at the boll stage. In 2015 and 2016, the proportions of the various hyperparasitoids were significantly different (2015: χ^2^ = 137.01, df = 21, *P* < 0.0001; 2016: χ^2^ = 82.81, df = 27, *P* < 0.0001). The hyperparasitoid species with the highest percentage varied among the different stages, with *S*. *taeniatus* (45.0%) highest at the seedling stage, *S*. *taeniatus*/*P*. *aphidis* (50.0%) highest at the bud stage, *P*. *aphidis* (58.8%) highest at the flowering stage and *A*. *suspensus* (56.0%) highest in 2015. In addition, *P*. *aphidis* led all the hyperparasitoids before the boll stage, with percentages over 45.0%, but *S*. *taeniatus* and *Syrphophagus* sp. made up 41.3% and 39.1%, respectively, ranking first instead of *P*. *aphidis* at boll stage in 2016 (Figs 3, 4, Table S1).

## Discussion

The cotton aphid has long been considered as an important pest in cotton fields^13^. As the parasitic natural enemies of aphids, aphid parasitoids, such as *Aphidius* spp., have been successfully used in the suppression of aphid populations^32^. Our study constitutes the first systematic description of *A*. *gossypii* parasitoid communities in the cotton agroecosystems of northern China, reporting a total of three primary parasitoid species and 12 hyperparasitoids. Parasitoids are important natural enemies of cotton aphids in northern China^14, 33^, with *Lysiphlebia japonica* widely assumed to be the dominant parasitoid species^18, 21, 23–29^. This parasitic wasp has been broadly studied in China, and applied research has quantified its impact on aphid population dynamics^33, 34^, its oviposition behaviour and preferences in terms of host instar^35^ and host plants^36^, and the effect of environmental conditions on its biological control efficacy^37^. Despite the wealth of laboratory trials and manipulative assays using *L*. *japonica*, it is rather surprising to note a complete absence of comprehensive, field-level records on *A*. *gossypii* parasitoid communities and aphid-parasitoid dynamics from cotton agroecosystems in northern China.

Our study reports a complete absence of *L*. *japonica* from some of China’s principal cotton-growing areas in Hebei, Beijing and Tianjin. Instead, *Binodoxys communis*, *Aphidius gifuensis* and *Aphelinus albipodus* were the three primary parasitoid species attacking cotton aphids at our research sites. The dominant species *B*. *communis* is indeed an effective parasitoid of *A*. *gossypii* ^38, 39^ but was earlier only recorded at low population levels in Shaanxi Province and the Shanghai region^27, 40^. Additionally, previous studies reported *A*. *gifuensis* as the dominant aphid parasitoid or natural enemy in cotton fields in northern China^14, 41^ and the Yellow River region^8^. Furthermore, our manuscript constitutes the first record of *A*. *albipodus* in Chinese cotton systems (except for Luo and Gan, 1986)^40^.

Hyperparasitoids represent an important trophic level in the aphid-parasitoid-hyperparasitoid community that should not be overlooked^18, 42, 43^. Nevertheless, few cotton aphid parasitoid studies have examined hyperparasitoids. In an investigation of the Aphidiidae in cotton fields in Jiangsu Province in southeast China, four hyperparasitoids were recorded. Only *Lygocerus koebelei* was identified to the species level, while others were indicated only by family (Pteromalidae and Figitidae) or genus (Aphidencyrtus [Syrphophagus]), and the corresponding host aphids were not recorded^10^. Such limited knowledge underrepresents the hyperparasitoids associated with cotton aphids in cotton fields in northern China. Our study found 12 hyperparasitoid species belonging to four families and six genera. Of these, *Pachyneuron aphidis* and species of *Syrphophagus* were the dominant hyperparasitoids. These results are very similar to those from our earlier hyperparasitoid surveys in wheat fields in northern China (unpublished data), except for those concerning the *Alloxysta* species, with *A*. *pusilla* being found in both wheat and cotton, while a new species, *A*. *brevis*, was found in this study in cotton.

Increases in parasitism levels were delayed relative to increases in aphids in this study. The highest parasitism rates were 12.3% in 2015 and 14.4% in 2016. The parasitism rate varies with the investigation time and site, ranging from 5 to 30%^21^. We found more hyperparasitoids at the seedling and bud stages, while there were more primary parasitoids in the late-season flowering and boll stages. This is the opposite result from that in the wheat fields in the earlier hyperparasitoid study, where hyperparasitoids occurred later in the growing season (unpublished data). Since cotton fields start growing after the wheat harvest in northern China, it would appear that hyperparasitoids in wheat fields might migrate to cotton to find new aphid hosts. Parasitoids often move through different habitats or crops in the agricultural landscape and over a range of distances^44^. By spraying rubidium chloride on wheat plants, *Dolichogenidea tasmanica* (Cameron) parasitoids could be tracked by that biological marker, and the appropriate flowering buckwheat, *Fagopyrum esculentum* Moench, could be deployed for biological control^45^. However, the uncultivated margins providing a resource for Aphidiinae parasitoids seemed to have a much more limited contribution than expected based on analysis with a molecular technique^46^. In the study of how the presence of maize (non-host plant) influences the movement of the parasitoid *Pediobius foveolatus* (Crawford) in the absence of hosts, the density of parasitoid wasps might primarily be determined by emigration rates^47^. The intercrops would be more effective in pest suppression if they are colonized by natural enemies before the pest-susceptible time^48^. Wheat-cotton intercropping preserved and augmented more parasitoids than cotton monoculture, especially when intercropping with an aphid-susceptible wheat variety, which was consistent with previous research results^14^. This migration phenomenon could explain the early occurrence of hyperparasitoids in cotton and the high similarity in the hyperparasitoid communities between wheat and cotton. Intercropping wheat and cotton fields may thus enhance the population size, survival, fecundity, longevity and behaviour of parasitoids and improve their control of cotton aphids^14, 49–51^.

Recently, emerging molecular techniques have made it easier to carry out in-depth studies of aphid-parasitoid interactions^42^ and have allowed us to better understand food web relationships^52^. To facilitate the establishment of a molecular detection system for cotton aphid parasitoids, knowledge of the parasitoid species involved is needed^42, 53^. This comprehensive survey of cotton aphid parasitoid species and composition allows for the establishment of such molecular detection techniques and the determination of the relative biological control effects of different parasitoids.

## Methods

### Study #1: Parasitoid species composition

#### Study area

This study was conducted in three provinces or cities (Hebei, Tianjin, and Beijing) of northern China. Twenty-three cotton fields were randomly selected in 2014 and 16 in 2015, and each field was visited and sampled three times. The location of each site was recorded using a handheld mobile GPS set (MG768W, Unistrong, Beijing, China). Cotton fields of >1 ha in size were randomly chosen, and individual fields were spaced at a minimum distance of 3.5 km (max. 75.4 km). Fields were planted in late April and managed by individual growers using crop management practices common in the region. Field sampling lasted from July 15^th^ to August 7^th^ in 2014 and from July 11^th^ to August 14^th^ in 2015 with intervals of 6–7 days between each sampling date in both years.

#### Sampling method

On each collection date, at least 150 aphid mummies were collected from five random locations within each field. Mummies were individualized in 1.5 mL centrifuge tubes and kept at 25 ± 1 °C, 65–75% RH and a 16:8 h L:D photoperiod until parasitoid emergence. Each tube was closed with an absorbent cotton ball. Parasitoid emergence was recorded on a daily basis, and wasps were stored in 75% ethanol at 4 °C for subsequent identification.

#### Species identification

Field-collected parasitoids were morphologically identified using Doğanlar (1986), Huang (1994), Shi and Shen (1995), Gibson and Vikberg (1998), Xiao and Huang (2000), Alekseev and Radchenko (2001), Chen and Shi (2001), Gibson (2001), Japoshvili (2007), Xiao *et al*. (2009), Rakhshani *et al*. (2012), and Ferrer-Suay *et al*. (2013a,b)^54–66^. For each parasitoid, key morphological features were observed and photographed using a polarizing microscope (DM2500, Leica, Germany) and a digital camera (EOS 505D, Canon, Japan).

### Study #2: Aphid-parasitoid seasonal dynamics

*Study area*. In late April 2015 and 2016, three replicate cotton field plots (var. SGK321) were planted at the Langfang Experimental Station (GPS coordinates 116.6°E, 39.5°N), Institute of Plant Protection (IPP) of the Chinese Academy of Agricultural Sciences (CAAS) in Langfang, Hebei Province. Each cotton field plot was 15 × 15 m and separated from the others by at least 5 m. All the field plots were located in the middle of large-area cotton fields (>1 ha) and at least 20 m away from other surrounding crops. No insecticides or herbicides were used, but all other common agronomic practices of northern China were applied, including regular inter-tillage, weeding and pruning. Prior to planting, all plots were fertilized with 375 kg/ha urea, 225 kg/ha phosphorus diamine, and 150 kg/ha potassium sulfate. One week after seedling emergence, 150 g/ha mepiquat chloride was applied.

#### Sampling method

Sampling was carried out every five days from May 30^th^ to September 7^th^ in 2015 and from June 4^th^ to September 12^th^ in 2016. During each sampling event, the number of cotton aphids and mummies were recorded on 20 cotton plants at each of five randomly selected sites per plot. Mummies were collected from the field and transported to the laboratory. Using the protocols described above, parasitoids were reared from each field-collected mummy and kept until further morphology-based identification.

### Data analysis

The species composition and the seasonal dynamics of the wheat aphids and parasitoids were investigated. The standard errors are presented in the figures as the error bars along with the mean number of aphids in the population. For study #2, a chi-squared test (proc freq) was performed to assess differences in the proportions of different parasitoid taxa, which included the two primary parasitoids and various hyperparasitoids, collected from cotton during different growing stages in 2015 and 2016. All analyses were performed using SAS 9.3 software (SAS Institute Inc., Cary, USA).

## Electronic supplementary material

Supplementary materials
