# Supplementary material for: Species composition and richness of aphid parasitoid wasps in cotton fields in northern China
Source: Sci Rep. 2017 Aug 29;7:9799. doi: 10.1038/s41598-017-10345-7 (PMC5575071; doi:10.1038/s41598-017-10345-7)
Supplement: Supplementary file 1 — Supplementary materials [file 41598_2017_10345_MOESM1_ESM.doc]

# Supplementary materials for

**Species composition and richness of aphid parasitoid wasps in cotton fields in northern China**

Fan Yang^1^, Yue-Kun Wu^1^, Lei Xu^1^, Qian Wang^1^, Zhi-Wen Yao^1^, Vladimir Žikić^2^, Željko Tomanović^3^, Mar Ferrer-Suay^4^, Jesús Selfa^4^, Juli Pujade-Villar^5^, Yan-Hui Lu^1^ & Yu-Yuan Guo^1^

^1^State Key Laboratory for Biology of Plant Diseases and Insect Pests, Institute of Plant Protection, Chinese Academy of Agricultural Sciences, Beijing 100193, China. ^2^Faculty of Sciences and Mathematics, Department of Biology and Ecology, University of Niš, Višegradska 33, 18000 Niš, Serbia. ^3^University of Belgrade, Faculty of Biology, Institute of Zoology, Department of Invertebrate Zoology and Entomology, Belgrade 11000, Serbia. ^4^Universitat de València, Facultat de Ciències Biològiques, Departament de Zoologia, València 46100, Spain. ^5^Universitat de Barcelona, Facultat de Biologia, Departament de Biologia Animal. Avda. Diagonal 645, 08028-Barcelona, Spain.

Correspondence and requests for materials should be addressed to Y.H.L (email: yhlu@ippcaas.cn) and Y.Y.G. (email: yuyuanguo@hotmail.com).

**Figure S1. The photos of** **key morphological identification characters of cotton aphid parasitoid species**

**(1) *Aphelimus albipodus***


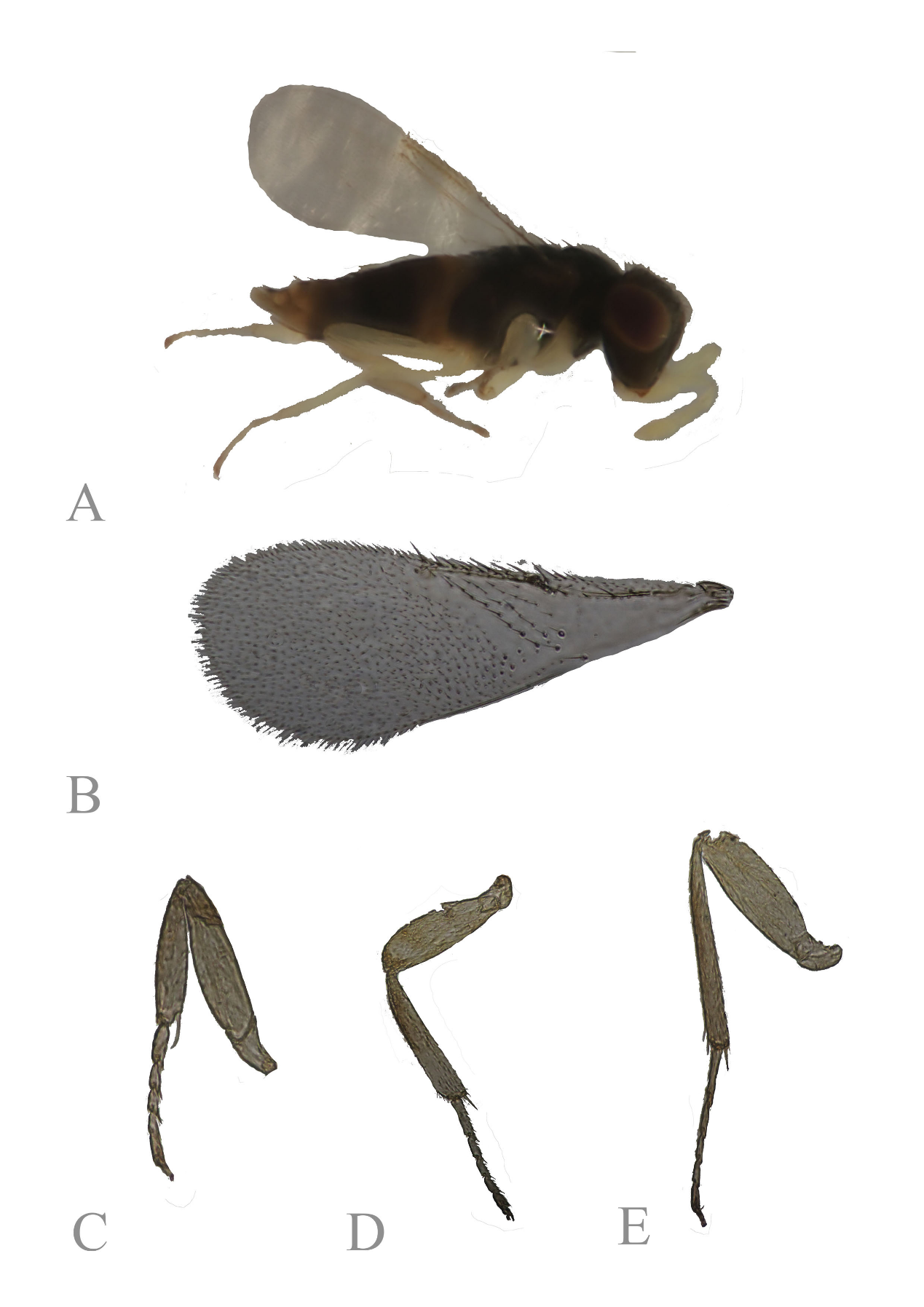


(A) Whole body, (B) forewing, (C) propodium, (D) mesopodium, (E) metapodium.

**(2) *Binodoxys communis***

**
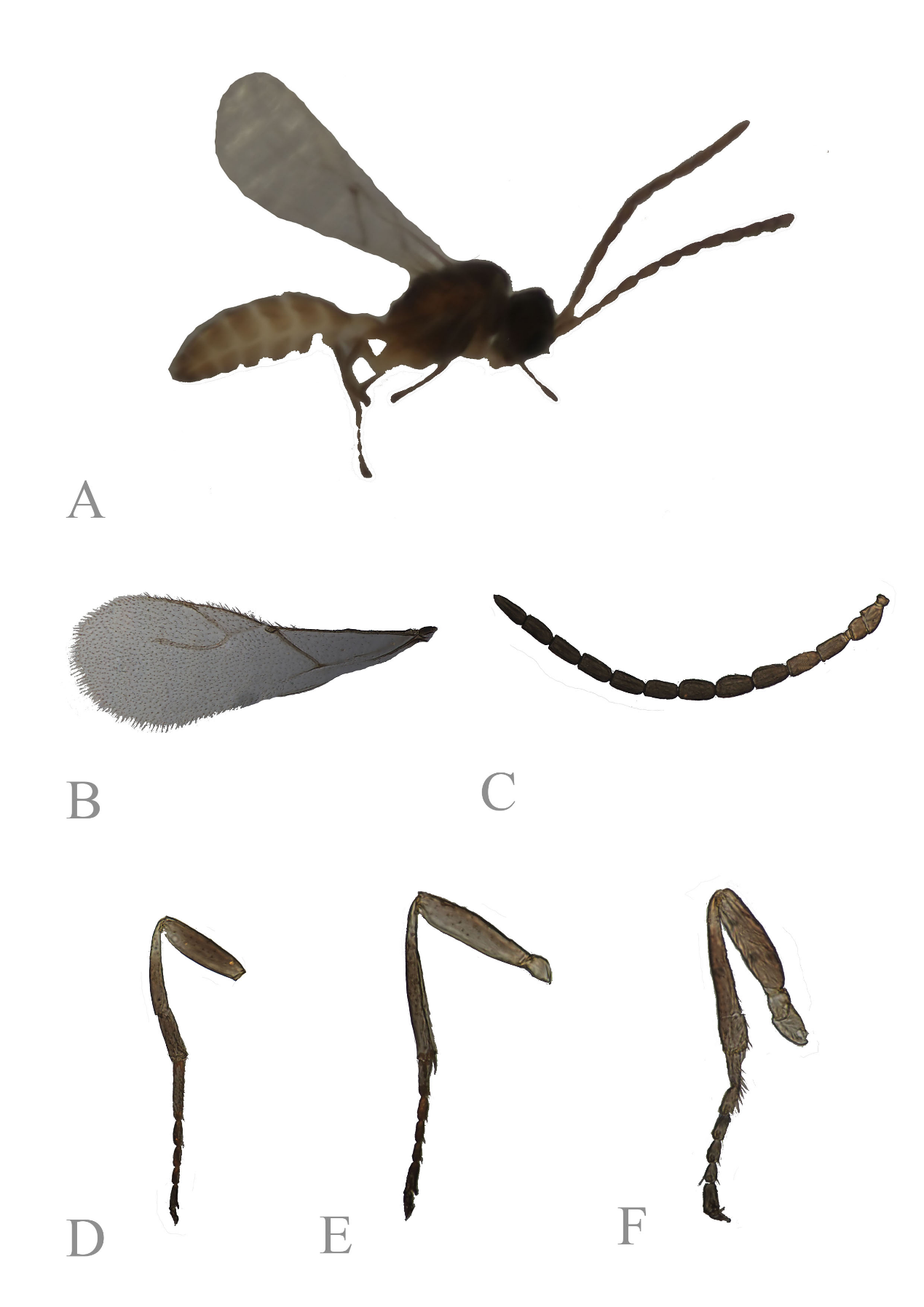
**

(A) Whole body, (B) forewing, (C) antenna, (D) propodium, (E) mesopodium, (F) metapodium.

**(3) *Alloxysta pusilla***


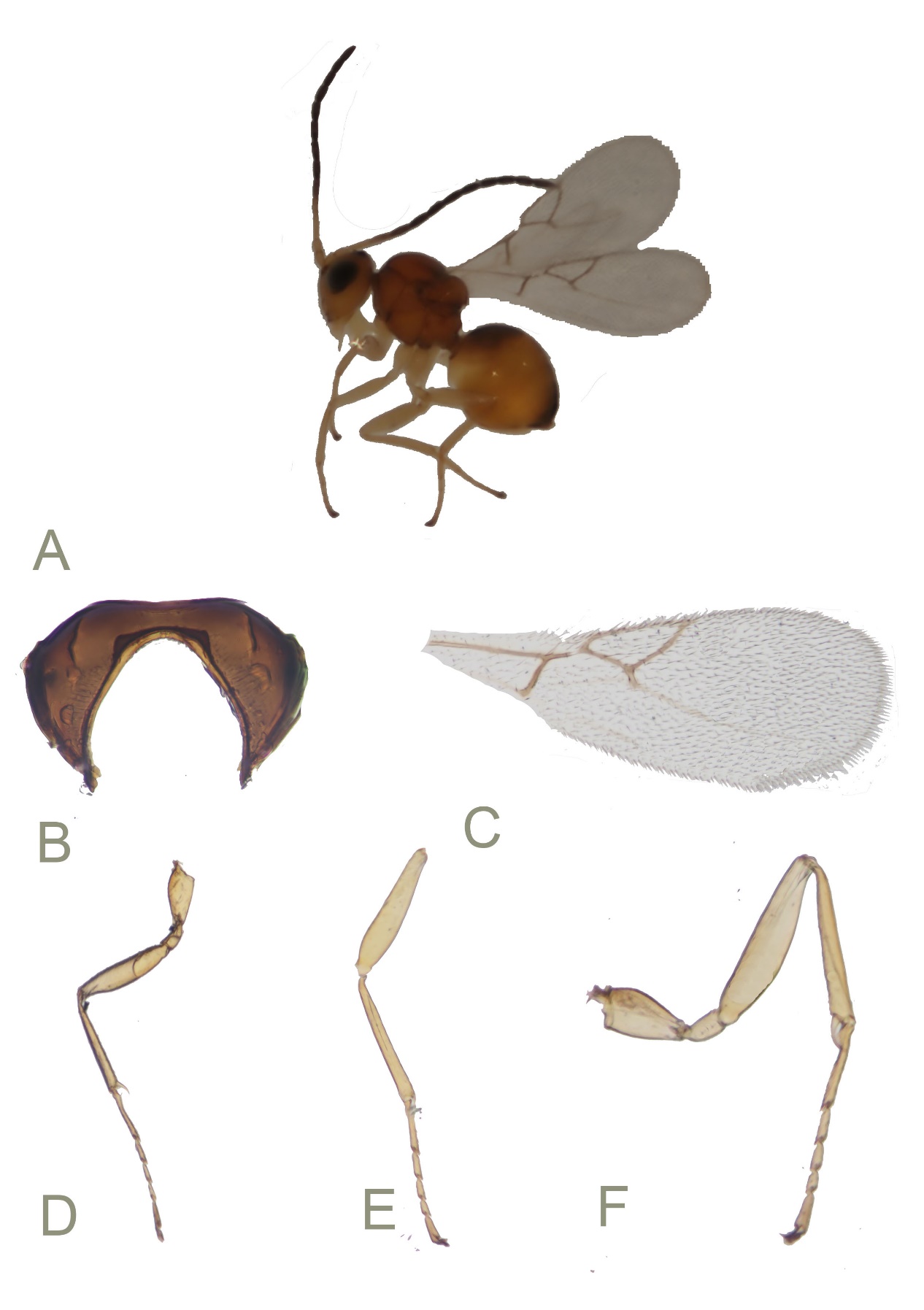


(A) Whole body, (B) pronotum, (C) forewing, (D) propodium, (E) mesopodium, (F) metapodium.

**(4) *Pachyneuron aphidis***


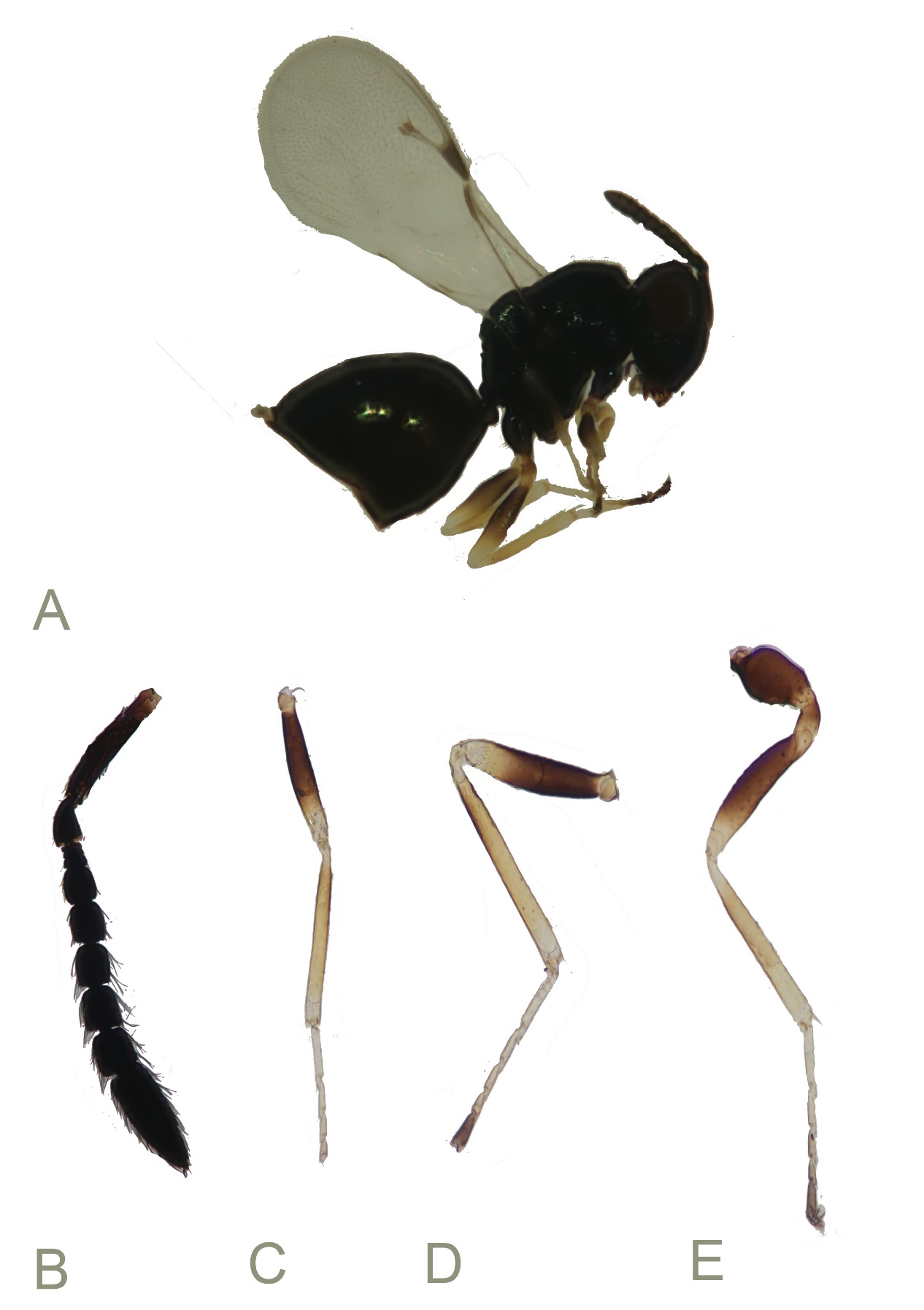


(A) Whole body, (B) antenna, (C) propodium, (D) mesopodium, (E) metapodium.

**(5) *Phaenoglyphis villosa***


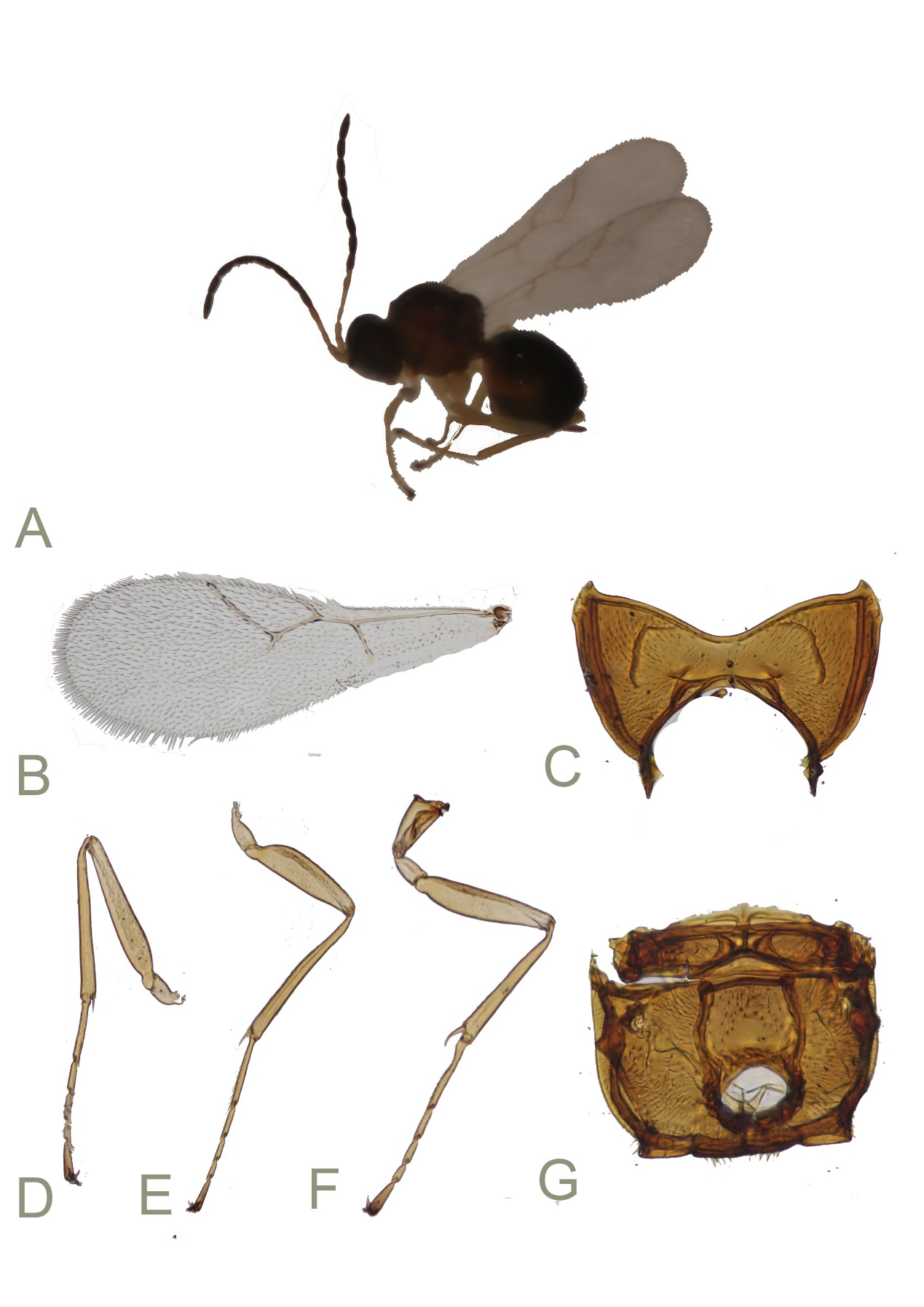


(A) Whole body, (B) forewing, (C) pronotum, (D) propodium, (E) mesopodium, (F) metapodium, (G) propodeum.

**(6) *Syrphophagus aphidivorus***


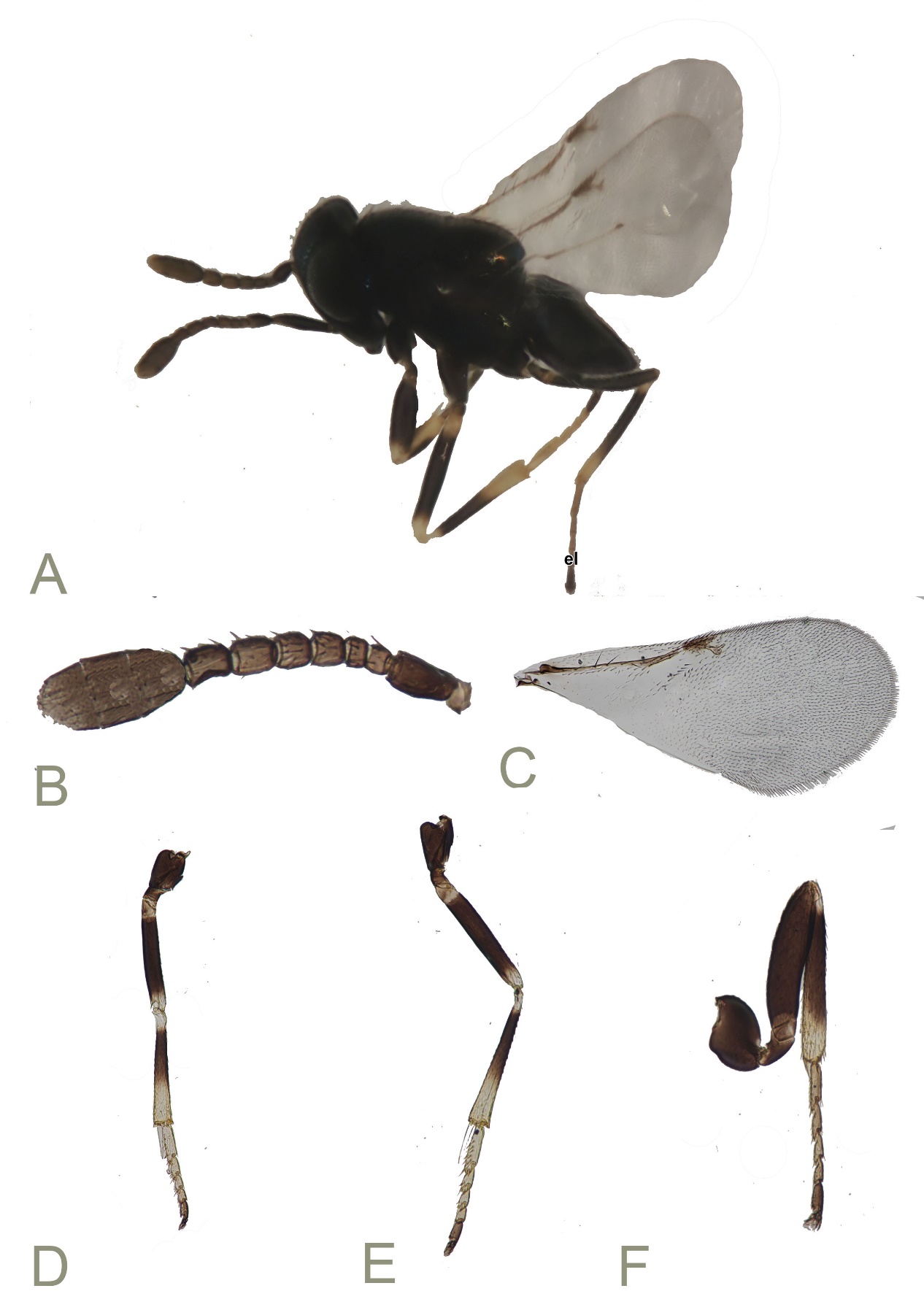


(A) Whole body, (B) antenna, (C) forewing, (D) propodium, (E) mesopodium, (F) metapodium.

**(7) *Syrphophagus eliavae***


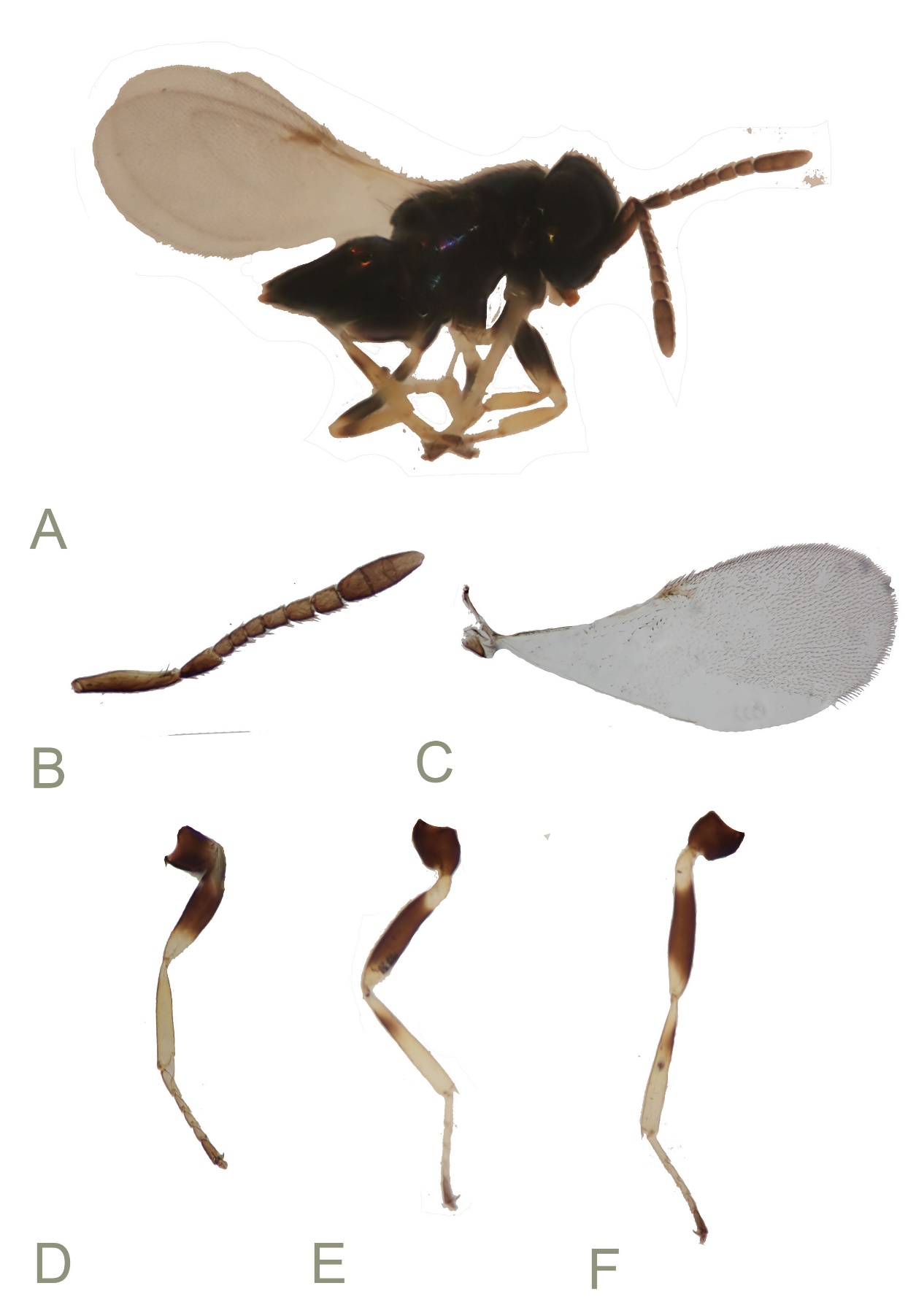


(A) Whole body, (B) antenna, (C) forewing, (D) propodium, (E) mesopodium, (F) metapodium.

**(8) *Syrphophagus* sp.**


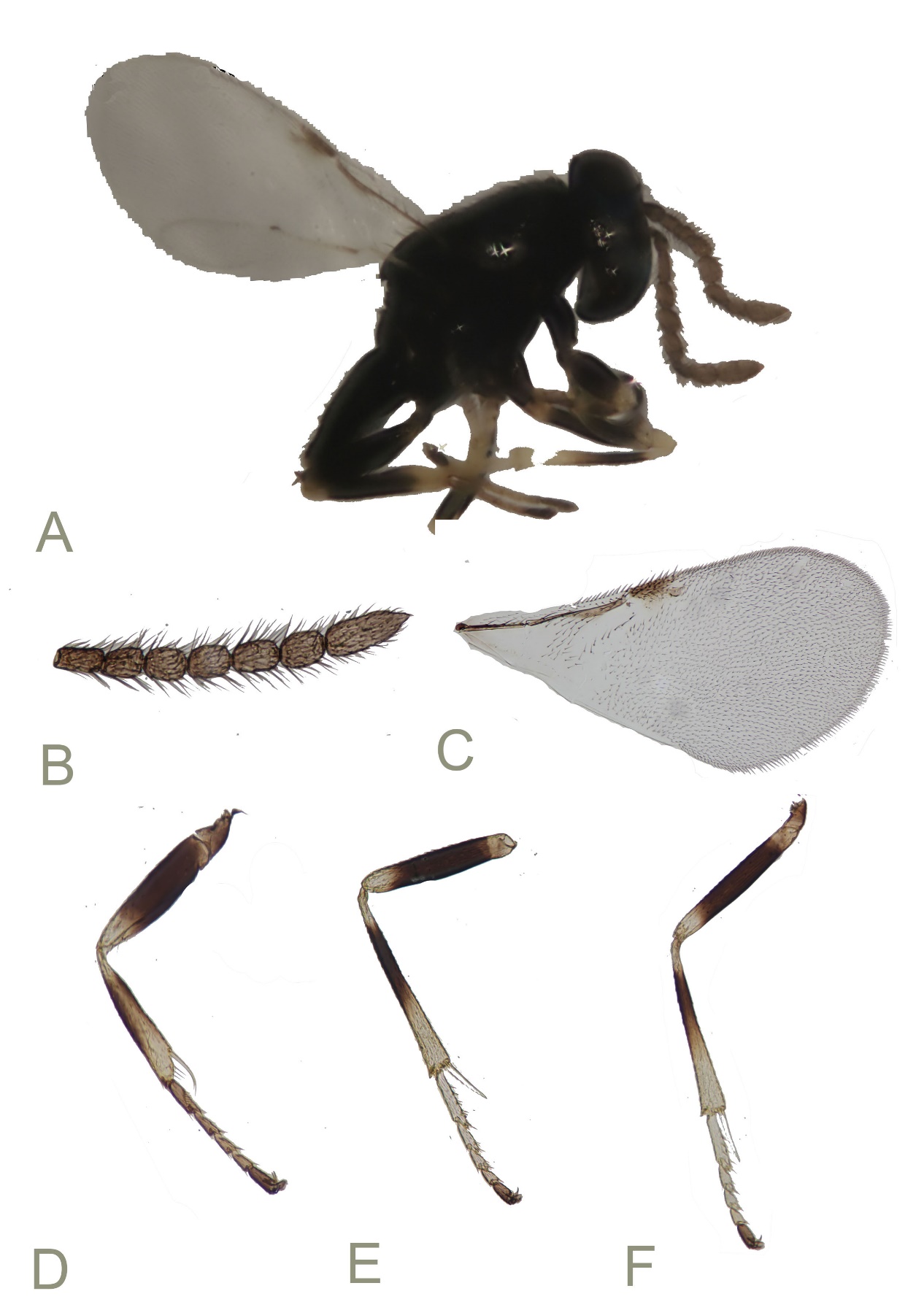


(A) Whole body, (B) antenna, (C) forewing, (D) propodium, (E) mesopodium, (F) metapodium, (G) propodeum.

**(9) *Syrphophagus taeniatus***


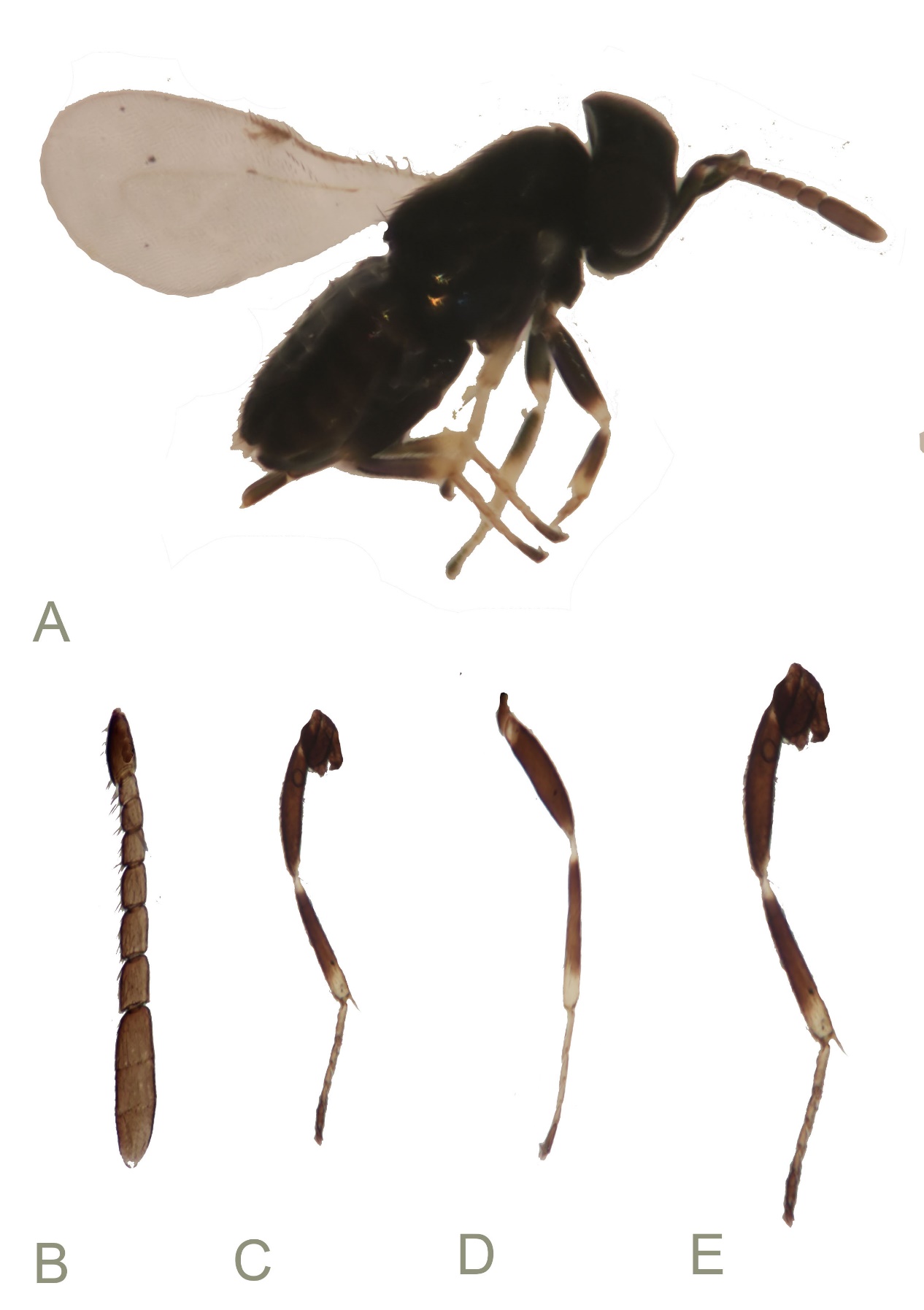


(A) Whole body, (B) antenna, (C) propodium, (D) mesopodium, (E) metapodium, (F) propodeum.

**(10) *Dendrocerus carpenteri***


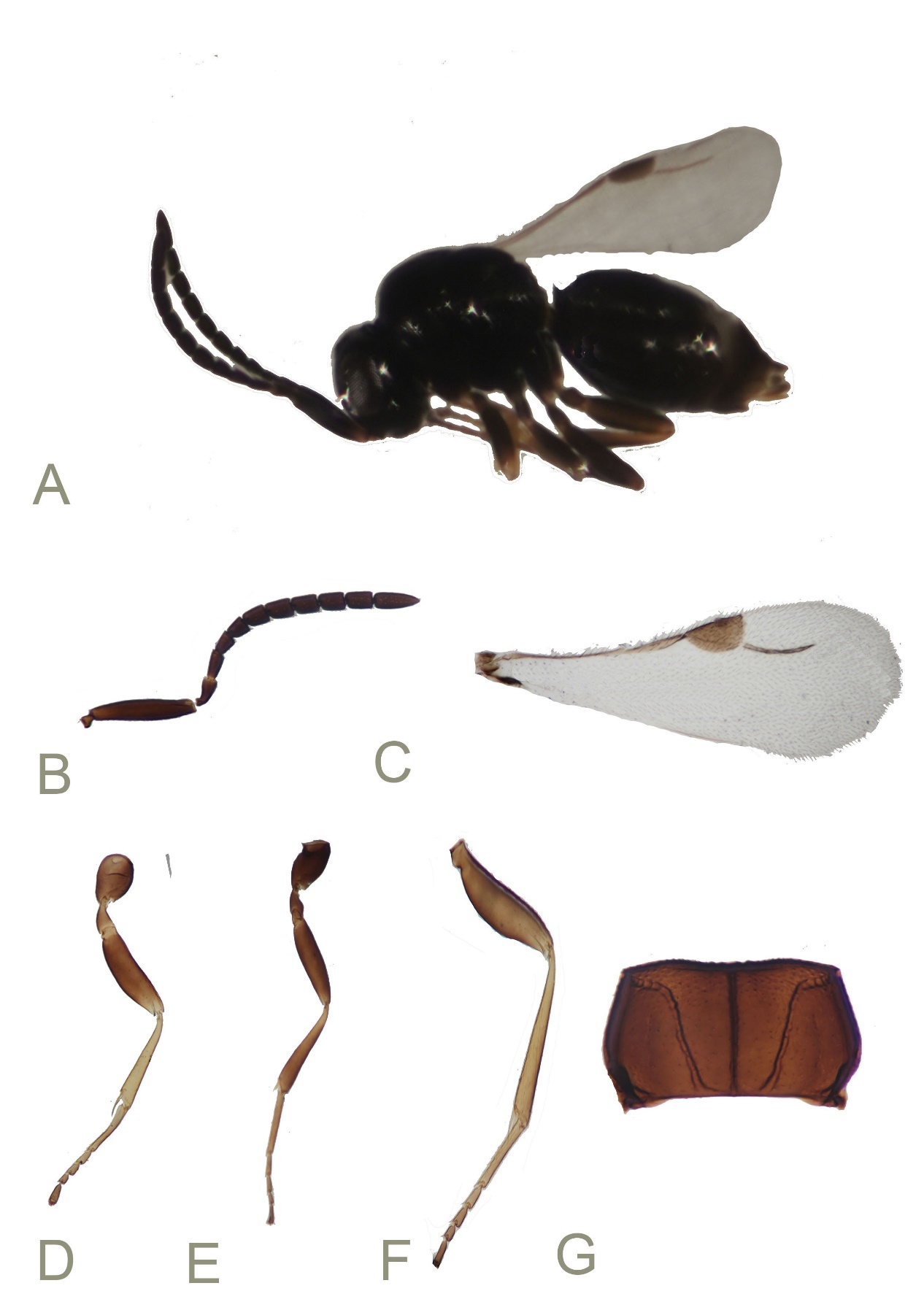


(A) Whole body, (B) antenna, (C) forewing, (D) propodium, (E) mesopodium, (F) metapodium, (G) mesonotum.

**(11) *Dendrocerus laticeps***


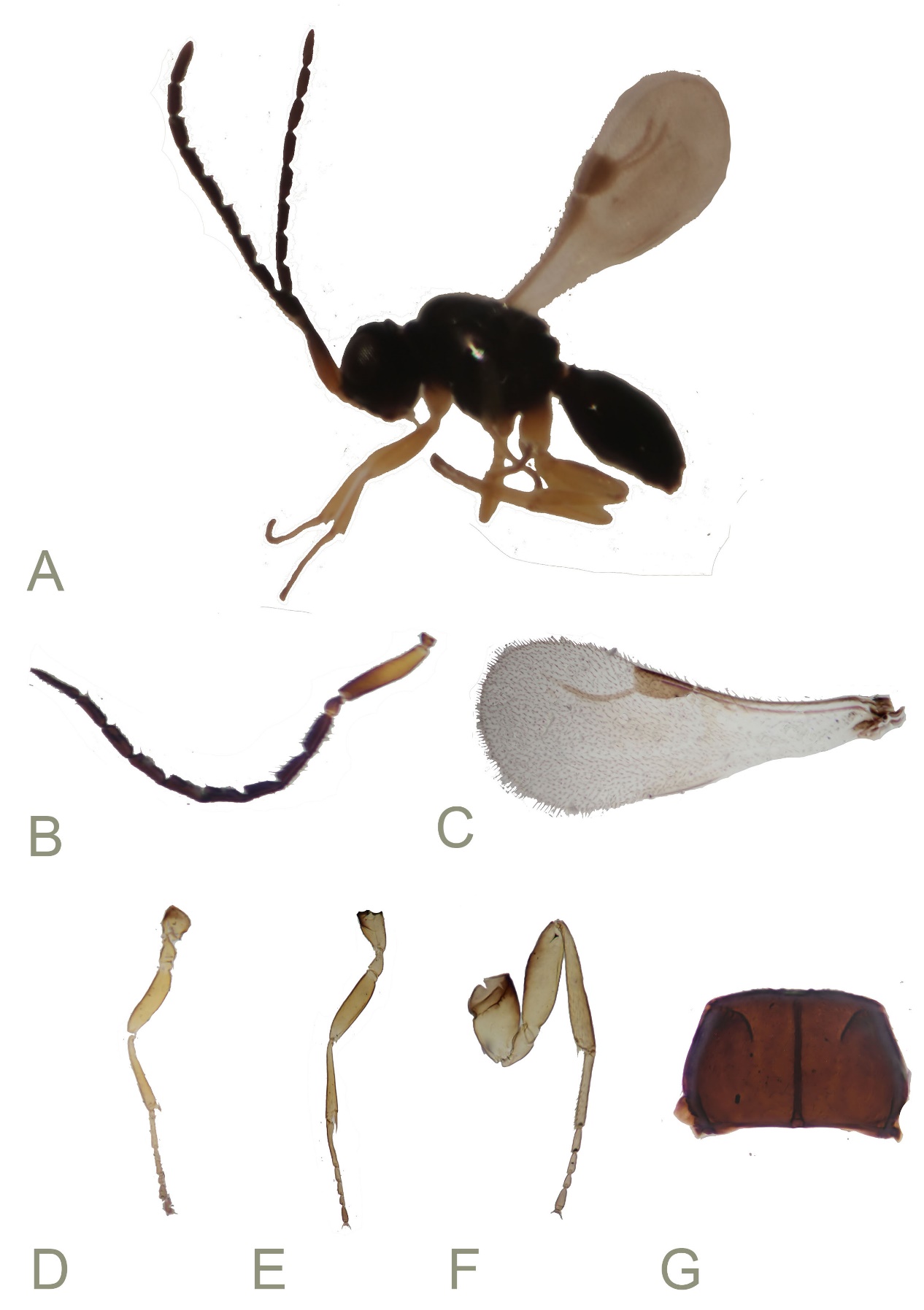


(A) Whole body, (B) antenna, (C) forewing, (D) propodium, (E) mesopodium, (F) metapodium, (G) mesonotum.

**(12) *Asaphes suspensus***


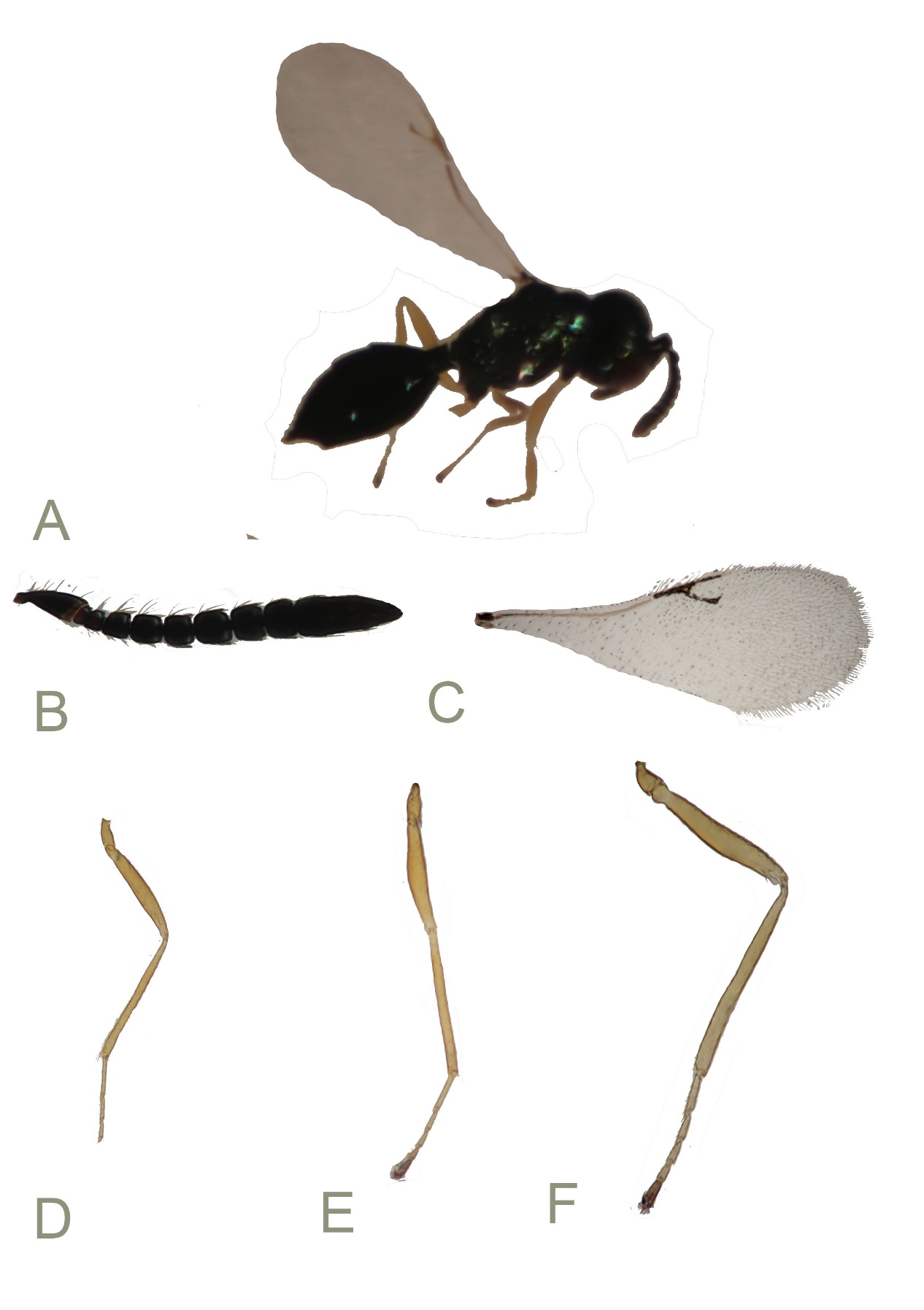


(A) Whole body, (B) antenna, (C) forewing, (D) propodium, (E) mesopodium, (F) metapodium.

**(13) *Asaphes vulgaris***


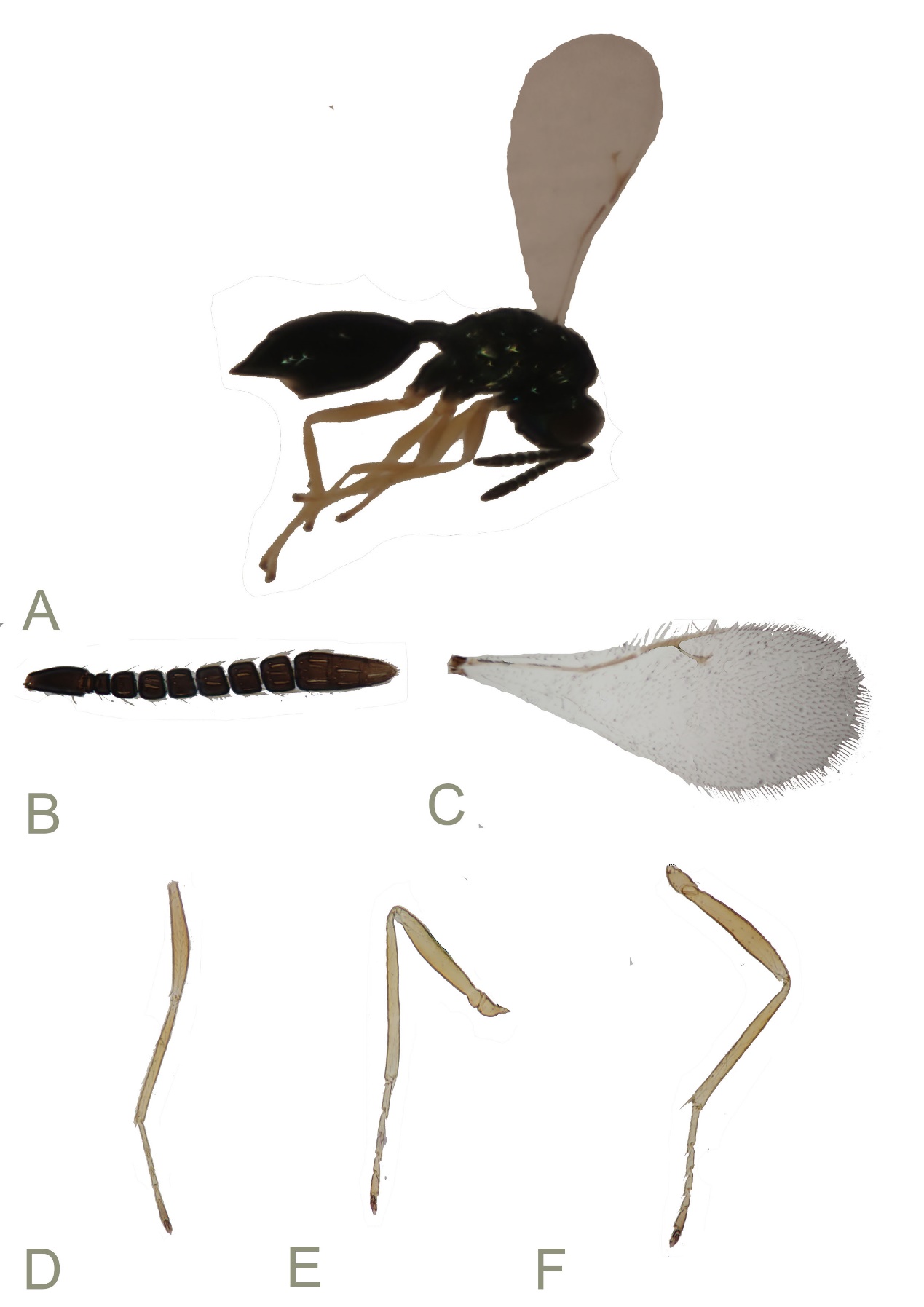


(A) Whole body, (B) antenna, (C) forewing, (D) propodium, (E) mesopodium, (F) metapodium.

**Table S1. The statistic analysis result of the difference of proportion among different parasitoid taxa that included the two primary parasitoids and various hyperparasitoids on cotton collected from different growing stages in 2015 and 2016.**

| **Year** | **Parasitoid groups** | **Sampling Period** | **df** | ***χ^2^*** | **P** |
| --- | --- | --- | --- | --- | --- |
| 2015 | Primary vs Hyper | The whole period | 3 | 16.49 | 0.0009 |
|  |  | Seeding vs Budding | 1 | 10.22 | 0.0014 |
|  |  | Seeding vs Flowering | 1 | 15.35 | <.0001 |
|  |  | Seeding vs Bollling | 1 | 4.49 | 0.0341 |
|  |  | Budding vs Flowering | 1 | 0.25 | 0.6146 |
|  |  | Budding vs Bolling | 1 | 1.23 | 0.2667 |
|  |  | Flowering vs Bolling | 1 | 1.26 | 0.2616 |
|  | Primary | The whole period | 3 | 0.71 | 0.8698 |
|  |  | Seeding vs Budding | 1 | 0.24 | 0.6210 |
|  |  | Seeding vs Flowering | --- | --- | --- |
|  |  | Seeding vs Bollling | --- | --- | --- |
|  |  | Budding vs Flowering | 1 | 0.12 | 0.7253 |
|  |  | Budding vs Bolling | --- | --- | --- |
|  |  | Flowering vs Bolling | 1 | 0.36 | 0.5465 |
|  | Hyper | The whole period | 21 | 137.01 | <.0001 |
|  |  | Seeding vs Budding | 3 | 0.51 | 0.9165 |
|  |  | Seeding vs Flowering | 3 | 15.80 | 0.0012 |
|  |  | Seeding vs Bollling | 7 | 90.97 | <.0001 |
|  |  | Budding vs Flowering | 3 | 1.97 | 0.5794 |
|  |  | Budding vs Bolling | 6 | 14.85 | 0.0215 |
|  |  | Flowering vs Bolling | 7 | 39.34 | <.0001 |
| 2016 | Primary vs Hyper | The whole period | 3 | 330.35 | <.0001 |
|  |  | Seeding vs Budding | 1 | 0.32 | 0.5704 |
|  |  | Seeding vs Flowering | 1 | 2.55 | 0.1106 |
|  |  | Seeding vs Bollling | 1 | 107.58 | <.0001 |
|  |  | Budding vs Flowering | 1 | 3.98 | 0.0460 |
|  |  | Budding vs Bolling | 1 | 247.94 | <.0001 |
|  |  | Flowering vs Bolling | 1 | 179.45 | <.0001 |
|  | Primary | The whole period | 3 | 103.97 | <.0001 |
|  |  | Seeding vs Budding | 1 | 5.03 | 0.0249 |
|  |  | Seeding vs Flowering | 1 | 6.21 | 0.0127 |
|  |  | Seeding vs Bollling | 1 | 192.66 | <.0001 |
|  |  | Budding vs Flowering | 1 | 0.03 | 0.8630 |
|  |  | Budding vs Bolling | 1 | 45.40 | <.0001 |
|  |  | Flowering vs Bolling | 1 | 43.00 | <.0001 |
|  | Hyper | The whole period | 27 | 82.81 | <.0001 |
|  |  | Seeding vs Budding | 5 | 5.98 | 0.3085 |
|  |  | Seeding vs Flowering | 6 | 8.33 | 0.2150 |
|  |  | Seeding vs Bollling | 6 | 32.27 | <.0001 |
|  |  | Budding vs Flowering | 7 | 29.28 | 0.0001 |
|  |  | Budding vs Bolling | 8 | 35.62 | <.0001 |
|  |  | Flowering vs Bolling | 8 | 40.31 | <.0001 |
